# Effect of Microwave Treatments Combined with Hot-Air Drying on Phytochemical Profiles and Antioxidant Activities in Lily Bulbs (*Lilium lancifolium*)

**DOI:** 10.3390/foods12122344

**Published:** 2023-06-12

**Authors:** Hong Quan, Yixi Cai, Yazhou Lu, Caifeng Shi, Xinghao Han, Linlin Liu, Xiu Yin, Xiaozhong Lan, Xinbo Guo

**Affiliations:** 1 Key Laboratory of Forest Ecology in Tibet Plateau, Ministry of Education, Tibet Agricultural and Animal Husbandry University, Nyingchi, Lhasa 860000, China; quanhong@xza.edu.cn; 2 School of Food Science and Engineering, South China University of Technology, Guangdong Province Key Laboratory for Green Processing of Natural Products and Product Safety, Engineering Research Center of Starch and Vegetable Protein Processing Ministry of Education, Guangzhou 510640, China; caiyixi2018@163.com (Y.C.); 13298124119@163.com (C.S.); 17876315457@163.com (L.L.); 3 The Provincial and Ministerial Co-Founded Collaborative Innovation Center for R & D in Tibet Characteristic Agricultural and Animal Husbandry Resources, The Center for Tibet Chinese (Tibetan) Medicine Resource, Joint Laboratory for Tibetan Materia Medica Resources Scientific Protection and Utilization Research of Tibetan Medical Research Center of Tibet, Tibet Agriculture and Animal Husbandry University, Nyingchi, Lhasa 860000, China; luyazhou@xza.edu.cn (Y.L.); hanxinghao@xza.edu.cn (X.H.); yinxiu@xza.edu.cn (X.Y.)

**Keywords:** lily bulbs, microwave treatment, phytochemicals, antioxidant activity, antiproliferation

## Abstract

Lily bulbs (*Lilium lancifolium* Thunb.) are rich in phytochemicals and have many potential biological activities which could be deep-processed for food or medicine purposes. This study investigated the effects of microwaves combined with hot-air drying on phytochemical profiles and antioxidant activities in lily bulbs. The results showed that six characteristic phytochemicals were identified in lily bulbs. They also showed that with an increase in microwave power and treatment time, regaloside A, regaloside B, regaloside E, and chlorogenic acid increased dramatically in lily bulbs. The 900 W (2 min) and the 500 W (5 min) groups could significantly suppress the browning of lily bulbs, with total color difference values of 28.97 ± 4.05 and 28.58 ± 3.31, respectively, and increase the content of detected phytochemicals. The highest oxygen radical absorbance activity was found in the 500 W, 5 min group, a 1.6-fold increase as compared with the control (57.16 ± 1.07 μmol TE/g DW), which was significantly relevant to the group’s phytochemical composition. Microwaves enhanced the phytochemicals and antioxidant capacity of lily bulbs, which could be an efficient and environmentally friendly strategy for improving the nutrition quality of lily bulbs during dehydration processing.

## 1. Introduction

Lily (*Lilium lancifolium* Thunb.) is a perennial herbaceous plant of the Liliaceae family whose bulbs are white and almost spherical. It is widely cultivated for its edible bulbs, which have been used for many centuries in East Asia as a food and traditional herb with a high nutritional and medicinal value. The bulbs are not only rich in nutrients such as carbohydrates, amino acids, dietary fibers, and mineral elements but also have abundant phytochemicals, such as phenolics, flavonoids, polysaccharides, and alkaloids [1]. Previous studies investigated the phytochemicals of lily bulbs and reported that phenolics and flavonoids as major phytochemical constituents exhibited diverse biological activities including antiviral, antitumor, antidiabetic, anti-inflammatory, and antioxidant activities and some potential health benefits [2]. The bulbs of the functional lily can be eaten fresh or deep-processed for industrial or medicinal purposes. Fresh-cut lily bulbs have broad consumption prospects in the vegetable market due to their high nutritional value and convenience in processing [3]. However, fresh lily bulbs are perishable and highly susceptible to turning brown in a short time if they are improperly handled, leading to a great loss of their nutritional and commercial value [4]. Therefore, exploring the appropriate processes to maintain the nutritional quality and medicinal value of lily bulbs would be the main task for lily bulbs' industrial application. 

Dried lily bulbs, as the dominant product in markets, can be easily subjected to long-term storage and conveniently consumed in these forms [4]. Several treatments have been used for lily bulb preservation, including heat treatment [5], radiation treatment [6], and chemical additive treatments [7]. However, radiation and chemical additive treatments also have many shortcomings, namely, difficulty in controlling operating conditions, high sensitivity of dosage to product quality, excessive residue of reagents, and so on [8]. The concerns about chemical residues have increased in the consumer markets, and exploring appropriate processing technologies for lily bulb preservation is extremely urgent. 

Drying is one of the most frequently used methods for lily bulb preservation, as reduced moisture content can hinder the growth and reproduction of microorganisms and minimize many moisture-mediated deteriorative reactions, including the loss of phytochemical compounds. The conventional hot-air drying method is widely applied in the industrialized production of agricultural materials due to the simple equipment, the diversified form of energy utilization, mass production, etc., which is also popularly practiced for lily bulb dehydration [9]. 

The quality attributes of products in terms of color, rehydration capacity, and appearance are affected by pretreatment, drying technology, as well as processing conditions employed [10]. Different drying methods have significant influences on phytochemical components, and regaloside A may be the main chemical component of lily bulbs [9]. The final products of lily bulbs are characterized by a yellowish-brown hue, nonuniform shrinkage, and the contamination of products. Significant color changes occur during hot-air drying [4]. Prolonged exposure to high air temperatures or long processing times can cause some undesirable quality changes in lily-bulb products including browning, oxidation, case hardening, and the degradation of nutrition and natural flavor [11]. Browning during the processing and storage of lily bulbs involves enzymatic and non-enzymatic browning, the former being mainly due to the oxidation of polyphenols by browning-related enzymes, including polyphenol oxidase (PPO) and peroxidase (POD), while the latter is mainly due to the carbonyl ammonia reaction between lily polysaccharide compounds and amino acids during storage and transport [12]. Browning can accelerate food spoilage and decay, affecting quality and flavor, while the color of lily bulbs is important for their acceptability. Therefore, in order to improve the drying process to maintain natural color and texture, and also to minimize nutrients and phytochemicals loss, the traditional hot-air drying methods may be improved by more efficient and advanced drying technologies for lily bulbs.

Microwaves have currently attracted lots of attention in the food industry due to their high-speed alternating electric fields, which can influence the interaction of food components [13]. Total phenolics increased significantly with microwave treatment in some fruit peels, and browning rates decreased with increased microwave treatment times except in the case of apple peels [14]. The interaction of food components can be also promoted under appropriate microwave treatment and leads to better efficacy in food processing. We hypothesized that microwave treatment combined with hot-air drying might significantly reduce the browning rate and improve the phenolic contents and total antioxidant activity in lily bulbs. Therefore, the objective of this study was to determine the effects of microwave treatment combined with hot-air drying on phytochemical profiles and antioxidant activities in lily bulbs. These results would provide the guidance for improving nutrition quality and drying efficiency of lily bulbs during dehydration processing.

## 2. Materials and Methods

### 2.1. Lily Bulbs Preparation and Microwave Treatment

The lily bulbs (*Lilium lancifolium* Thunb.) were cultivated in the same field at Tibet Agricultural and Animal Husbandry University’s Research Base, Linzhi, Tibet, China. Fresh lily bulbs were collected in the early spring before budding and washed with tap water to remove the residual dirt and soil on the surface. Then, the lily bulbs’ surfaces were dried, and the lily bulbs were peeled to small scales. The small scales of a hundred lily bulbs (approx. 5 kg) were evenly divided into ten groups (approx. 500 g per group) for the subsequent microwave treatment and hot-air drying experiments. An inverter microwave oven was used for fresh lily bulb pretreatments. The microwave pretreatment conditions were set at 2 min with changed powers as 100 wattages (W), 300 W, 500 W, 700 W, and 900 W and rated power at 500 W with changed times as 1 min, 3 min, 4 min, and 5 min. Fresh lily bulbs were used as a non-microwave pretreatment control group (CK). After microwave pretreating, all ten lily-bulb groups were dried at 60 °C with an air-dry oven until the moisture level dropped below 1% for subsequent analysis. 

### 2.2. Determination of Color Difference Values 

The color difference of ten samples of lily bulbs was determined by the software Image J (National Institutes of Health, Bethesda, MD, USA). The color difference values of three parameters were measured to characterize the color of lily bulbs: the brightness value L*, with a larger L* meaning a higher brightness (otherwise, it is darker); the redness–greenness value a*, with −a* meaning green and +a* meaning red; and the yellowness–blueness value b*, with −b* indicating blue and +b* indicating yellow. The total color difference values were calculated using the following formula:

$$\Delta E=\sqrt{({L-L_{0})}^{2}+({a-a_{0})}^{2}+({b-b_{0})}^{2}}$$

### 2.3. Extraction and Determination of Phytochemicals 

Total phenolics of lily bulbs were extracted in accordance with the previous method [15] with some modifications. In brief, dry samples were milled into powder, and 1.0 g was extracted with 10 mL of 80% methanol solution by means of ultrasound (100 kW). After centrifugation, the supernatant was used for further analysis. All the experiments were run in three replicates. Total phenolic contents were determined using the Folin-Ciocalteu assay. Results of phenolic content were expressed as milligrams of gallic acid equivalent per 100 g dry weight (mg GAE/100 g DW). Phytochemical profiles were performed by Waters HPLC-PAD (photodiode array detector) system (Waters Corporation, Milford, MA, USA) as described previously [16]. The mobile phases were 0.1% trifluoroacetic acid in water (A) and acetonitrile (B) with gradient elution program. The gradient profile was 0–5 min from 10 to 17% B, 5–8 min from 17 to 20% B, 8–12 min 20 to 24% B, 12–15 min 24 to 28% B, 15–20 min 28 to 95% B, 20–21 min 95 to 10% B, and from 21 to 23 min, solvent B was kept at 10%. The wavelength of detection was set at 320 nm and the total flow rate was 1 mL/min. The characteristic peaks of lily bulb extracts were determined by the related standards. Data were presented as micrograms per gram in dry weight (μg/g DW).

### 2.4. Antioxidant Activities in Lily Bulbs

The determination of the antioxidant activities in lily bulbs included 1,1-diphenyl-2-picrylhydrazyl (DPPH) radical scavenging capacity, 2,2′-azinobis-(3-ethylbenzthiazoline-6-sulphonate) (ABTS) radical scavenging capacity, and oxygen radical absorbance capacity (ORAC). DPPH method was conducted as reported previously [17], the absorbance was measured with Microplate Reader (Molecular Devices, Sunnyvale, CA, USA) at 517 nm, and the value was calculated by means of the standard ascorbic acid (ASA). DPPH value was expressed as micromole ASA equivalent per gram in dry weight (μmol ASA/g DW) in triplicates. ABTS method was carried out with a T-AOC Assay Kit (Beyotime Biotechnology, Shanghai, China), the absorbance was measured at 734 nm, and the value was calculated using Trolox as standard. Data were expressed as micromole Trolox equivalent per gram in dry weight (μmol TE /g DW) in triplicates. ORAC method was conducted as described before [17]. The decay of fluorescence for 35 cycles every 4.5 min at the excitation of 485 nm and the emission of 535 nm was determined. Trolox was used as standard and the absorbance was measured with a FilterMax F5 Multi-Mode Microplate Reader (Molecular Devices, Sunnyvale, CA, USA). Data were expressed as micromole Trolox equivalent per gram in dry weight (μmol TE/g DW) in triplicates.

### 2.5. Determination of Antiproliferation and Cytotoxicity

The antiproliferation and cytotoxicity assays were performed using the methylene blue colorimetric method as previously reported [16]. The HepG2 cell line (ATCC: HB-8065) was purchased from American Type Culture Collection (ATCC, Manassas, VA, USA). HepG2 cells were invariably cultured at 37 °C and 5% CO_2_ in William’s Medium E (WME) supplemented with 5% FBS (fetal bovine serum), 10 mM HEPES, 100 μg/mL gentamicin, 0.05 μg/mL hydrocortisone, 2 mM L-glutamine, 5 μg/mL insulin, 50 units/mL penicillin, and 50 μg/mL streptomycin. HepG2 cells in WME were inoculated on 96-well microplates at densities of 2.5 × 10^4^ cells/well (cytotoxicity) or 1.5 × 10^4^ cells/well (antiproliferative activity) and cultured at 37 °C for 4 h or 24 h. The medium was replaced by a growth medium which contained lily bulbs extract at different concentrations, and then the cells were cultured at 37 °C for 24 h (cytotoxicity) and 72 h (antiproliferative activity). The absorbance of the reaction solution in each well was measured at 595 nm using Microplate Reader (Molecular Devices, Sunnyvale, CA, USA), and the final number of living cells per well was calculated using the methylene blue colorimetric method. The antiproliferative activity was calculated using IC_50_ values, and the cytotoxicity was calculated using CC_10_ values and expressed as mg per mL dry weight (mg/mL DW) with three replicates.

### 2.6. Statistical Analysis

Significance analysis was conducted through one-way analysis of variance (ANOVA) and Tukey multiple comparison test (*p* < 0.05) by using IBM SPSS 25.0 (SPSS Inc., Chicago, IL, USA). Pearson correlation was analyzed by Origin 2018 (Origin Lab Corporation, Northampton, MA, USA). Results were expressed as mean ± SD for triplicates.

## 3. Results

### 3.1. Effect of Microwave Pretreatment for Color Changes in Lily Bulbs 

**Figure 1:**
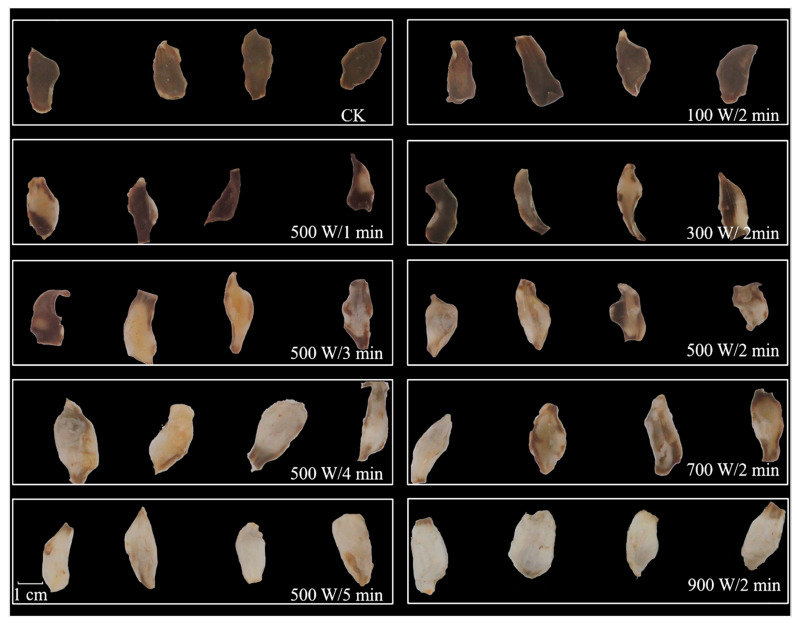
Color changes in lily bulbs with microwave pretreatments.

The lily bulbs’ color changes with different microwave pretreatments were shown in Figure 1, and the color difference values were shown in Table 1. In the microwave power groups, the L* values ranged from 36.76 ± 7.01 to 64.14 ± 1.94. They were all larger than the CK group (35.56 ± 2.12) and showed a tendency to increase with increasing power. The a* values were all smaller than the CK group (4.43 ± 0.30), except for the 500 W, 2 min group (5.08 ± 0.83). The maximum value of b* was found in the 500 W, 3 min group (12.84 ± 1.06) and was not significantly different from the CK group (12.35 ± 1.14). The ΔE values ranging from 5.04 ± 0.96 to 28.97 ± 4.05 were positively correlated with microwave power. In the treatment time groups, the L* value varied from 32.24 ± 2.86 to 64.00 ± 1.37. They were in line with the trend of the microwave power group and were greater than the CK group except for the 500 W, 1 min group (32.24 ± 2.86). The a* value ranging from 2.08 ± 0.31 to 6.02 ± 0.15 tended to decrease gradually over time. The maximum value of b* occurred in the 500 W, 4 min group (12.89 ± 0.68) and was not significantly different from the control group. The ΔE values ranging from 7.04 ± 2.41 to 28.58 ± 3.31 were positively correlated with microwave treatment time.

### 3.2. Effect of Microwave Pretreatment for Phenolic Content of Lily Bulbs 

**Figure 2:**
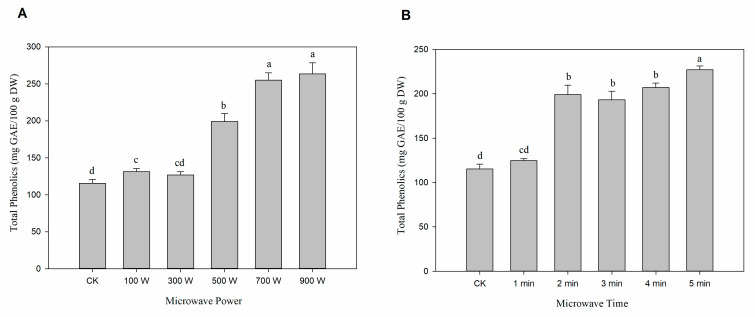
Changes of phenolic contents in lily bulbs with microwave pretreatments. (**A**) Fixed treatment time to 2 min with changed microwave power. (**B**) Fixed microwave power to 500 W with changed treatment times. Different letters (a–d) on each column indicate a significant difference at *p* < 0.05.

The effect of the microwave pretreated on phenolic contents in lily bulbs was presented in Figure 2. The results showed that the phenolic content increased with microwave power from 100 to 900 W and time from 1 to 5 min. The range of phenolic content in the microwave power and time groups were 126.72 ± 4.3 to 263.34 ± 14.99 and 124.85 ± 2.03 to 227.25 ± 4.1 mg GAE/100 g DW, respectively, and all of them were greater than the CK group (115.35 ± 5.44 mg GAE/100 g DW). 

Regaloside A, regaloside B, regaloside C, regaloside E, Chlorogenic acid as well as *p*-coumaric acid were identified and quantified by HPLC-PAD in Table 2. Regaloside A, regaloside B, regaloside E, and chlorogenic acid were the main components of the phenolic composition of lily bulbs, and all showed an increasing trend with increasing microwave power and time. Compared to the CK group (228.6 ± 14.7μg/g DW), regaloside A increased significantly by 13.39–398.69% due to microwave treatment, with the highest at 900 W, 2 min group (1023 ± 20μg/g DW) and 500 W, 5 min group (1140 ± 16μg/g DW). Vary from regaloside A, regaloside B, regaloside E, and chlorogenic acid were 471.8 ± 7.5, 111.4 ± 1.2 and 128.5 ± 7.5μg/g DW in 100 W, 1 min group, respectively, each smaller than their CK group (519.1 ± 11.8, 256.4 ± 26.9, 305.2 ± 11.3 μg/g DW). As with regaloside A, regaloside B, regaloside E, and chlorogenic acid achieved maximum values in the microwave power group at 900 W, 2 min, 2.53, 7.8, and 4.3 times higher than the respective control groups, respectively, and in the microwave time group at 500 W, 5 min, 2.9, 7.2, and 3.2 times higher than the respective control groups, respectively. Of the six phenolic components, *p*-coumaric acid is the least abundant phenolic compound in lily bulbs, accounting for 1.07% to 3.16% of the total. The *p*-coumaric acid content was significantly and positively correlated with microwave power and time. Thus, the maximum contents were also found in the 900 W, 2 min group (61.44 ± 2.42 μg/g DW) and the 500 W, 5 min group (58.08 ± 4.36 μg/g DW), with no significant difference. Compared to the CK group (432.8 ± 13.5 μg/g DW), microwave treatment caused a 4.3–33.29% declination of regaloside C, except for 500 W, 5 min group (488.7 ± 6.0 μg/g DW). The increase of microwave power induced an increase (100–500 W) and then a decline (500–900 W) in regaloside C content, reaching a maximum value in the 500 W, 2 min group (400.4 ± 10.1 μg/g DW). Regaloside C tended to increase with increasing microwave time, except for the 500 W, 4 min group, and reached a maximum in the 500 W, 5 min group.

### 3.3. Effect of Microwave Pretreatment for Antioxidant Activity in Lily Bulbs

The antioxidant activities in lily bulbs are presented in Table 3, expressed as the DPPH value, ABTS value, and ORAC value. The results showed that after microwave pretreatment, the DPPH values of lily bulbs decreased in comparison to the control group (4.85 ± 0.26 μmol ASA/g DW). The range of DPPH values for microwave power and time groups were 2.66 ± 0.43 to 4.10 ± 0.14 and 1.50 ± 0.02 to 1.91 ± 0.52 μg ASA/g DW, respectively. In the microwave power group, the highest value was found in the 300 W, 2 min group (4.10 ± 0.14 μmol ASA/g DW), but there was no significant difference between the microwave time groups. ABTS values were not significantly different from the control group (6.03 ± 0.72 μmol TE/g DW), except for the 900 W, 2 min group. The ABTS values for the microwave power and time group ranged from 4.78 ± 0.06 to 6.99 ± 0.33 and 5.06 ± 1.26 to 6.03 ± 0.72 μmol TE/g DW. The maximum values in these two groups were found in the 500 W, 3 min and 700 W, 2 min groups, with no significant difference. As the minimum value of ABTS for the different groups, the 900 W, 2 min group was 94.47% of the 500 W, 1 min group. In contrast with DPPH, ORAC values were generally above those of the control group (57.16 ± 1.07 μmol TE/g DW). The ranges of ORAC values were 26.17 ± 1.01 to 76.39 ± 2.11 and 32.04 ± 0.99 to 93.75 ± 5.12 μmol TE/g DW individually for the microwave power and time group. The ORAC values of the microwave power groups went up with increasing microwave power from 100 W to 900 W. For the microwave time groups, the highest ORAC value was found in the 5 min group with an increase of 64.01%, followed by the 3 min group (59.87%) and the 4 min group (24.58%), but the 1 min group had a decrease of 43.95%. Based on the above data, the 500 W, 5 min groups had the highest ORAC activity.

### 3.4. Effect of Microwave Pretreatment for Cell Antiproliferation and Cytotoxicity in Lily Bulbs

The changes in antiproliferative activities and the cytotoxicity of lily bulb extracts with different microwave pretreatments were shown in Table 4. The cytotoxicity of the lily bulb extracts was expressed as CC_10_, CC_20,_ and CC_50_ (mg/mL). The data indicated that there were positive relationships between the concentration of the extracts of lily bulbs and the cytotoxicity effect. Particularly, the 100 W, 2 min group and the 500 W, 1 min group showed maximum cytotoxicity to HepG2 cells than others. It was found that the CC_50_ value of the extracts under different treatment conditions is greater than the IC_50_ value, which means that all the lily bulb extracts have potential antiproliferative activity in concentrations that are nontoxic to HepG2 cells. The half-maximal inhibitory concentration (IC_50_) was used to express the cell antiproliferation of lily bulb extracts. The IC_50_ value of the lily bulb extracts decreases considerably with an increase in microwave power from 300 to 900 W and an increase in time from 3 to 5 min. For different microwave power, it was found that microwave pretreatment at 100 W for 2 min had the strongest inhibitory effect and the lowest IC_50_ (21.17 ± 0.36 mg/mL), followed by 900 W (30.16 ± 1.28 mg/mL). From different microwave times, the IC_50_ value of the 5 min and 500 W treatment is the lowest.

**Figure 3:**
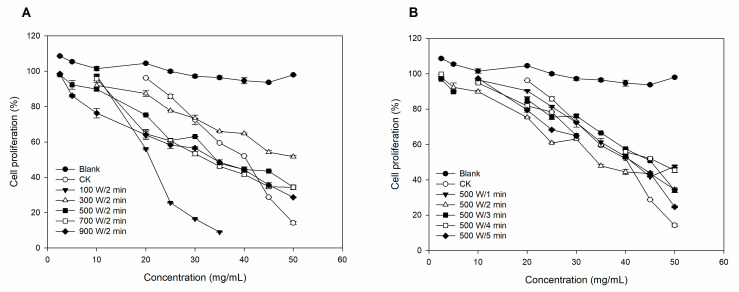
Changes of antiproliferative activities in lily bulbs with microwave pretreatments. (**A**) Fixed treatment time to 2 min with changed microwave power. (**B**) Fixed microwave power to 500 W with changed treatment times.

The antiproliferative effects on HepG2 cells of lily bulb extracts were shown in Figure 3. It showed that the extract significantly inhibited the growth of HepG2 cells in a concentration-dependent manner. At a concentration of 10 mg/mL, the significant inhibitory effect of the lily bulb extracts started. As shown in the figures, microwave power (Figure 3A) has a greater effect on cell antiproliferative activity than microwave time (Figure 3B). The 100 W and 2 min treatment groups have significant antiproliferative activity in the whole group, and the inhibition rate has reached 90.96% at a concentration of 35 mg/mL. The antiproliferative activity in the 900 W, 2 min group was slightly higher than the control group, inhibiting growth by 71.32% at 50 mg /mL, whereas that of the 500 W, 5 min group reached 75.35%.

### 3.5. Correlation Analysis

In order to determine the effect of the phytochemical composition of the lily bulb on the color and antioxidant activity studied, a simple correlation analysis was carried out. Table 5 exhibited the correlation analysis results among color, total phenolic contents, phytochemical compounds, and antioxidant activity in lily bulb extracts. The total phenolic contents and phenolics, except for regaloside C, are the main factors that significantly affect the △E value. There was no significant correlation among regaloside C, the △E value, the DPPH value, and the ABTS value. The ORAC value was found significantly relevant to total phenolic contents (*p* < 0.05), regaloside A (*p* < 0.01), regaloside B (*p* < 0.01), regaloside E (*p* < 0.01), Chlorogenic acid (*p* < 0.05) and *p*-coumaric acid (*p* < 0.01) whereas the DPPH value was significantly negatively correlated with them. It was also found that there was no significant correlation between the antioxidant activities assays.

## 4. Discussion

### 4.1. Effect of Microwave Pretreatment for Browning of Lily Bulbs

The results presented in this manuscript demonstrate a significant increase in the L* value and ΔE value of lily bulbs with increasing microwave power from 300 to 900 W and time from 3 to 5 min (Table 1). It had been noted that changes in the brightness of dried samples can serve as a measure of browning [18]. Thus, the observed increase in the L* value may indicate that microwave pretreatment is effective in reducing the browning of lily bulbs. The phenomenon can be attributed to the inactivation of endogenous browning-related enzymes such as PPO and POD, which are responsible for the browning response of lily bulbs [4]. Therefore, the 900 W, 2 min group and 500 W, 5 min group had the best conditions for microwave pretreatment and inhibiting the browning of lily bulbs. These conditions were found to be effective due to their relatively high heating efficiency, resulting in more color degradation associated with microwave pretreatment. Furthermore, this may be related to the increased content of regaloside A, regaloside B, regaloside E, chlorogenic acid, and *p*-coumaric acid during microwave pretreatment, as the L* values are positively correlated with them (Table 5). It is worth noting that chlorogenic acid has been shown to be involved in apple browning by accelerating the formation of 5-hydroxymethyl furfural [19].

### 4.2. Effect of Microwave Pretreatment for Phenolic Content of Lily Bulbs

It was found that the content of total phenolics, regaloside A, regaloside B, regaloside E, chlorogenic acid and *p*-coumaric acid increased but the content of regaloside C decreased with increasing microwave power from 300 to 900 W and time from 3 to 5 min in lily bulbs (Table 2). We found that the 900 W, 2 min group had optimal conditions for microwave pretreatment, and the 500 W, 5 min group had optimal conditions for increasing the phenolic content of lily bulbs. This is likely due to the fact that microwaves generate immense heat and pressure inside the cells, causing them to rupture rapidly. This process releases soluble phenolics from the plant matrix while also damaging those phenolics that are sensitive to heat due to the high amount of energy generated [20]. However, the molecular mechanism of phytochemical changes with microwave treatment has not been engaged. Further research should be carried out on how microwave treatments combined with hot-air drying affect the other phytochemicals in lily bulbs, which might provide a deeper understanding of the action mechanisms behind those phenomena.

### 4.3. Effect of Microwave Pretreatment for Bioactivity in Lily Bulbs

Lily bulbs contain a wealth of nutrients and various bioactive substances, such as polysaccharides, saponins, and colchicine, which show a wide range of biological and pharmacological activities, antioxidant and antitumor effects, etc. Consequently, the quality of lily bulbs pretreated with a microwave under different conditions can be assessed by measuring the extract’s biological activity. Screening of biological samples for antioxidant capacity is generally performed using a series of assays rather than relying on a single assay that may not provide realistic results [21]. Therefore, we evaluated the antioxidant activity in the lily bulb extract using three different assays (namely, DPPH, ABTS, and ORAC) to obtain a comprehensive picture of its actual antioxidant role. As Figure 3 illustrates, we found that the 500 W, 5 min group had the highest ORAC values, followed by the 500 W, 3 min group and the 900 W, 2 min group, all of which were higher than the control group. These results were generally consistent with the data from the previous experiments, which showed that the 500 W, 5 min group had the highest content of phenolics. Furthermore, there is a significant correlation between the phytochemicals and the ORAC value on the basis of correlation analysis. Although all the lily bulb extracts showed antioxidant potential in the ORAC, DPPH, and ABTS assays, these activities did not correlate significantly with the TPC values. It is supposed that the presence of varying amounts of polysaccharides or other secondary metabolites from lily bulb extract exerts greater antioxidant potentials that mask the effects of phenolics or that the specific phenolic species present in the system are not adequately quantifiable [12,22]. According to reports in the published literature, polysaccharides, which make up 10–36% of the dry weight of the bulb, are one of the richest carbohydrate components in the lily and have potent antioxidant activities as assessed by DPPH [23,24]. A recent study showed a significant positive correlation between some alkaloids, terpenoids, and other substances in lily, which may be important antioxidants [25]. Therefore, the identification and quantification analysis of other constituents should be considered in follow-up studies to obtain a comprehensive picture of the antioxidant activity in lily bulb extract and associated components. 

Phytochemical extracts from fruits or vegetables have been shown to have strong antiproliferative activity in many studies. Several bioactive constituents of lily bulbs, such as phenols, saponins [26], polysaccharides [27], and colchicine [28] have been found to have certain antitumor activities. As illustrated in Table 4 and Figure 3, the 900 W, 2 min treatment group has the second most effective cell antiproliferative activity after the 100 W, 2 min group. At the same concentration, the cell survival rate of the 900 W, 2 min group was lower than that of the control group, indicating that the inhibition of HepG2 cell proliferation was enhanced under this pretreatment condition. However, according to the results of the HPLC detection, we were unable to find a positive correlation between the content of the detected phytochemical components and the antiproliferative activity in the lily bulb extract. Based on the above study, lily bulbs are a valuable source of bioactive compounds with potent antiproliferative activity against HepG2 cells. However, the synergistic effect between different constituents present in the extract may be responsible for this activity, rather than the phenolics alone. This is also the reason why the 100 W, 2 min group had particularly significant cytotoxicity, so we speculate that under the conditions of low power and short time microwave, alkaloids, saponins, and other secondary metabolites are still present in large quantities [29]. Further research is needed to fully understand the complex interactions between different lily bulb components.

## 5. Conclusions

In the present study, we investigated the different influences on color, phytochemical profiles, and bioactivities in lily bulbs with various microwave parameters. The results showed that six characteristic phytochemicals were identified in lily bulbs. Regaloside A, regaloside B, regaloside E, and chlorogenic acid were dramatically increased in lily bulbs with an increase in microwave power and treatment time. Among these compounds, the maximum values were observed in the 900 W, 2 min group, which were 2.53, 7.8, and 4.3 times higher than the respective control groups. Similarly, in the 500 W, 5 min group, these compounds showed increased levels of 2.9, 7.2, and 3.2 times that of the control groups. The 900 W, 2 min and the 500 W, 5 min groups could significantly suppress the browning of lily bulbs, with total color difference values of 28.97 ± 4.05 and 28.58 ± 3.31, respectively, and increase the content of detected phytochemicals. The highest oxygen radical absorbance activity was found in the 500 W, 5 min group (a 1.6-fold increase as compared with the control (57.16 ± 1.07 μmol TE/g DW), which is significantly relevant to phytochemical composition). However, the 900 W, 2 min group showed better antiproliferation activity than other groups without cytotoxicity with an IC_50_ value of 21.17 ± 0.36 mg/mL. Microwave pretreatment significantly enhanced the phytochemical contents and antioxidant capacity of lily bulbs and inhibited bulb browning and HepG2 cell proliferation during hot-air drying. Microwave treatment is a well-established method for food processing, but there are no reports of its combination with hot-air drying for lily bulbs. Meanwhile, most research on microwave technology focuses mainly on moisture content, with little attention paid to the study of the active compounds represented by polyphenols. This study indicates that pretreatment with a microwave could be developed as an efficient and environmentally friendly strategy for improving the nutrition quality of lily bulbs during dehydration processing. 

## Figures and Tables

**Table 1:** Changes in color difference values of lily bulbs with microwave pretreatments.

Treatments	L* Value	a* Value	b* Value	ΔE Value
CK	35.56 ± 2.12 cd	4.67 ± 0.70 b	12.35 ± 1.14 ab	0.00 ± 0.00
100 W/2 min	38.13 ± 2.03 c	4.43 ± 0.30 bc	8.94 ± 0.96 de	5.04 ± 0.96 e
300 W/2 min	36.76 ± 7.01 cd	3.19 ± 0.40 d	8.90 ± 2.69 de	5.73 ± 1.32 e
500 W/2 min	48.44 ± 2.79 b	5.08 ± 0.83 b	12.84 ± 1.06 a	12.90 ± 1.84 d
700 W/2 min	53.42 ± 2.45 b	3.69 ± 0.29 cd	12.62 ± 0.58 ab	17.92 ± 0.93 c
900 W/2 min	64.14 ± 1.94 a	0.65 ± 0.29 f	10.13 ± 0.99 cd	28.97 ± 4.05 a
500 W/1 min	32.24 ± 2.86 d	6.02 ± 0.15 a	6.60 ± 2.13 e	7.04 ± 2.41 e
500 W/3 min	50.26 ± 2.74 b	6.02 ± 0.40 a	12.77 ± 1.30 a	14.91 ± 2.12 cd
500 W/4 min	58.91 ± 1.19 a	3.10 ± 0.29 d	12.89 ± 0.68 a	23.41 ± 0.99 b
500 W/5 min	64.00 ± 1.37 a	2.08 ± 0.31 e	12.60 ± 0.94 ab	28.58 ± 3.31 a

L* means black and white color difference, values from 0 to 100, 0 being black and 100 being white. a* means redness and greenness color difference, negative value being greenness and positive value being redness. b* means yellowness and blueness color difference, negative value being blueness and positive value being yellowness. ΔE value means total aberration. Different letters (a–e) within each column indicate a significant difference at *p* < 0.05.

**Table 2:** Changes of phytochemical contents (μg/g DW) in lily bulbs with microwave pretreatments.

Treatments	Regaloside A	Regaloside B	Regaloside C	Regaloside E	Chlorogenic Acid	*p*-Coumaric Acid
CK	228.6 ± 14.7 h	519.1 ± 11.8 f	432.8 ± 13.5 b	256.4 ± 26.9 h	305.2 ± 11.3 h	36.49 ± 4.55 c
100 W/2 min	259.2 ± 4.2 g	471.8 ± 7.5 g	288.7 ± 3.1 f	111.4 ± 1.2 i	128.5 ± 7.5 i	14.03 ± 3.08 e
300 W/2 min	359.3 ± 3.2 f	646.9 ± 7.7 e	311.7 ± 6.5 e	546.4 ± 11.0 g	324.3 ± 6.8 g	24.05 ± 7.06 d
500 W/2 min	922.9 ± 19.1 de	1138 ± 19 d	400.4 ± 10.1 c	1281 ± 20 d	767.6 ± 2.0 e	38.12 ± 1.99 bc
700 W/2 min	956.0 ± 20.5 c	1231 ± 8 c	382.7 ± 8.8 d	1232 ± 20 e	1034 ± 12 b	44.94 ± 2.43 b
900 W/2 min	1023 ± 20 b	1312 ± 29 b	385.2 ± 8.2 d	2006 ± 41 a	1313 ± 4 a	61.44 ± 2.42 a
500 W/1 min	350.5 ± 7.6 f	666.7 ± 13.7 e	303.2 ± 4.5 e	640.9 ± 23.1 f	348.8 ± 8.8 f	20.55 ± 2.32 d
500 W/3 min	944.2 ± 17.1 cd	1219 ± 1 c	414.2 ± 9.1 c	1617 ± 39 c	756.2 ± 11.1 e	42.64 ± 4.76 bc
500 W/4 min	907.5 ± 32.3 e	1158 ± 16 d	378.5 ± 9.0 d	1289 ± 45 d	834.8 ± 5.8 d	44.49 ± 1.79 b
500 W/5 min	1140 ± 16 a	1504 ± 15 a	488.7 ± 6.0 a	1838 ± 22 b	988.0 ± 12.3 c	58.08 ± 4.36 a

Different letters (a–i) within each column indicate a significant difference at *p* < 0.05.

**Table 3:** Changes of antioxidant activities in lily bulbs with microwave pretreatments.

Treatments	DPPH Value (μmol ASA/g DW)	ABTS Value (μmol TE/g DW)	ORAC Value (μmol TE/g DW)
CK	4.85 ± 0.26 a	6.03 ± 0.72 ab	57.16 ± 1.07 de
100 W/2 min	3.58 ± 0.19 c	6.01 ± 1.47 abc	26.17 ± 1.01 g
300 W/2 min	4.10 ± 0.14 b	5.78 ± 0.15 abc	34.67 ± 1.34 f
500 W/2 min	2.66 ± 0.43 c	6.68 ± 0.53 a	60.89 ± 1.37 d
700 W/2 min	2.86 ± 0.16 c	6.99 ± 0.33 a	55.10 ± 1.34 e
900 W/2 min	2.77 ± 0.43 c	4.78 ± 0.06 c	76.39 ± 2.11 b
500 W/1 min	1.76 ± 0.03 e	5.06 ± 1.26 bc	32.04 ± 0.99 f
500 W/3 min	1.50 ± 0.02 e	6.34 ± 0.62 a	91.38 ± 1.48 a
500 W/4 min	1.76 ± 0.35 e	5.93 ± 1.59 abc	71.21 ± 3.20 c
500 W/5 min	1.91 ± 0.52 e	6.03 ± 0.72 ab	93.75 ± 5.12 a

Different letters (a–g) within each column indicate a significant difference at *p* < 0.05.

**Table 4:** Changes of antiproliferative activities and cytotoxicity in lily bulbs with microwave pretreatments.

Treatments	Antiproliferation IC_50_ (mg/mL)	Cytotoxicity (mg/mL)		
		CC_10_	CC_20_	CC_50_
CK	37.26 ± 0.28 c	54.84 ± 0.62 cd	63.26 ± 0.08 f	80.75 ± 0.62 f
100 W/2 min	21.17 ± 0.36 f	34.37 ± 0.15 e	39.90 ± 0.38 g	51.47 ± 0.15 g
300 W/2 min	55.55 ± 5.78 a	73.88 ± 0.56 a	88.16 ± 0.69 b	119.2 ± 0.6 b
500 W/2 min	36.47 ± 0.93 cd	57.14 ± 0.53 bcd	72.64 ± 0.27 d	109.5 ± 0.5 c
700 W/2 min	33.07 ± 1.56 de	71.18 ± 0.41 a	83.49 ± 0.65 c	109.7 ± 0.4 c
900 W/2 min	30.16 ± 1.28 e	59.02 ± 1.09 b	81.62 ± 2.81 c	142.1 ± 1.1 a
500 W/1 min	45.92 ± 1.41 b	57.06 ± 1.18 bcd	69.99 ± 0.31 e	99.25 ± 1.18 e
500 W/3 min	56.23 ± 2.30 a	54.23 ± 0.80 d	69.68 ± 0.82 e	106.9 ± 0.8 d
500 W/4 min	44.06 ± 0.30 b	71.68 ± 2.10 a	92.65 ± 3.67 a	143.7 ± 2.1 a
500 W/5 min	36.93 ± 0.26 c	57.69 ± 0.47 bc	73.50 ± 0.44 d	111.2 ± 0.5 c

Different letters (a–g) within each column indicate a significant difference at *p* < 0.05.

**Table 5:** Pearson correlation coefficient among color, total phenolic contents, phytochemical compositions, and antioxidant activity with microwave pretreatments.

	L	a	b	△E	TPC	Reg A	Reg B	Reg C	Reg E	Chl	ρ-cou	DPPH	ABTS	ORAC
L	1	−0.683 *	0.583	0.968 **	0.922 **	0.923 **	0.922 **	0.608	0.898 **	0.920 **	0.904 **	−0.500	0.146	0.802 **
a		1	−0.112	−0.684 *	−0.576	−0.427	−0.450	−0.247	−0.479	−0.594	−0.633 *	−0.090	0.248	−0.316
b			1	0.393	0.526	0.603	0.579	0.797 **	0.446	0.456	0.628	−0.123	0.705 *	0.727 *
ΔE				1	0.899 **	0.909 **	0.920 **	0.493	0.920 **	0.927 **	0.854 **	−0.618	−0.005	0.731 *
TPC					1	0.928 **	0.916 **	0.488	0.884 **	0.977 **	0.850 **	−0.48	0.210	0.669 *
Reg A						1	0.993 **	0.600	0.955 **	0.936 **	0.838 **	−0.663 *	0.301	0.807**
Reg B							1	0.640 *	0.966 **	0.937 **	0.866 **	−0.665 *	0.271	0.831 **
Reg C								1	0.568	0.498	0.792 **	−0.177	0.428	0.865 **
Reg E									1	0.944 **	0.869 **	−0.672 *	0.049	0.829 **
Chl										1	0.886 **	−0.535	0.072	0.711 *
ρ-cou											1	−0.341	0.093	0.878 **
DPPH												1	−0.009	−0.499
ABTS													1	0.233
ORAC														1

Notes: TPC: total phenolic content; Reg A: regaloside A; Reg B: regaloside B; Reg C: regaloside C; Reg E: regaloside E; Chl: Chlorogenic acid; *p*-cou: *p*-coumaric acid. * and ** mean significant correlation at the 0.05 and 0.01 levels, respectively (2-tailed).

## Data Availability

The data used to support the findings of this study can be made available by the corresponding author upon request.
